# Video assisted thoracic surgery vs. thoracotomy for locally advanced lung squamous cell carcinoma after neoadjuvant chemotherapy

**DOI:** 10.1186/s13019-018-0813-7

**Published:** 2018-12-17

**Authors:** Likui Fang, Luming Wang, Yiqing Wang, Wang Lv, Jian Hu

**Affiliations:** Department of Thoracic Surgery, the First Affiliated Hospital, Zhejiang University School of Medicine, Hangzhou, 310003 China

**Keywords:** Locally advanced lung squamous cell carcinoma, Neoadjuvant chemotherapy, Video assisted thoracic surgery, Thoracotomy

## Abstract

**Background:**

Surgery is an important part of multidisciplinary treatment strategy for locally advanced lung squamous cell carcinoma (LSCC), but insufficient evidence supports the feasibility and safety of video assisted thoracic surgery (VATS) following neoadjuvant chemotherapy for locally advanced LSCC. This study aims to compare perioperative data and long-term survival of locally advanced LSCC patients between VATS and thoracotomy after neoadjuvant chemotherapy.

**Methods:**

We retrospectively collected the clinical and pathological information of patients with locally advanced LSCC who underwent surgical resection after neoadjuvant chemotherapy from October 2013 to October 2017. All patients were divided into two groups (thoracotomy and VATS) and were compared the differences in perioperative, oncological and survival outcomes.

**Results:**

A total of 81 patients were analyzed in this study (67 thoracotomy and 14 VATS). VATS provided less postoperative pain (*P* = 0.005) and produced less volume of chest drainage (*P* = 0.019) than thoracotomy, but the number of resected lymph nodes was less in VATS group (*P* = 0.011). However, there was no significant difference in the number of resected lymph node stations and the rate of nodal upstaging between two groups. The mean disease free survival (DFS) was 32.7 ± 2.7 months for the thoracotomy group and 31.8 ± 3.0 months for the VATS group (*P* = 0.335); the corresponding overall survival (OS) was 41.7 ± 2.2 months and 36.4 ± 4.1 months (*P* = 0.925).

**Conclusion:**

In selected patients with locally advanced LSCC, VATS played a positive role in postoperative recovery and associated similar survival outcome compared with thoracotomy after neoadjuvant chemotherapy.

## Introduction

Lung cancer is one of the most common cancers and the leading cause of cancer-related death in the world, and more than 80% of patients have a group of histological subtypes known as non-small cell lung cancer (NSCLC) [1]. Lung adenocarcinoma (LA) and lung squamous cell carcinoma (LSCC) are the most common subtypes of NSCLC [2]. Although operable early stage NSCLC has satisfactory prognosis with the improvement of medical technology [3, 4], the 5-year survival rate of locally advanced NSCLC (LANSCLC) with surgery alone is only 20–35% [5]. So far, the treatment of LANSCLC has evolved from surgery alone to multidisciplinary pattern. It has been proven that neoadjuvant chemotherapy could significantly improve overall survival, time to distant recurrence, and recurrence-free survival [6], while preoperative radiotherapy do not add any survival benefit to neoadjuvant chemotherapy followed by surgery [7]. Neoadjuvant chemotherapy has been a valid treatment option for most of patients with LANSCLC. However, there also have been many controversial debates about the operation based multidisciplinary treatment, one of which is the selection of surgical approaches after neoadjuvant chemotherapy, thoracotomy or video assisted thoracic surgery (VATS).

VATS is superior to open surgery for the resection of early stage NSCLC, because it can minimize complications, provide less pain and offer faster recovery with at least equivalent long-term survival rate [8, 9]. VATS was initially proposed only for operable early stage lung cancer, but in recent years it has been carried out by some experienced thoracic surgeons in LANSCLC with satisfactory outcome [10, 11]. However, there were only few studies reporting the outcome of VATS following neoadjuvant therapy [12, 13] and currently, no published study reported the comparison between VATS and thoracotomy following neoadjuvant chemotherapy in locally advanced LSCC staged by the eighth American Joint Committee on Cancer (AJCC 8) staging system.

In this study, we aimed to compare perioperative data and long-term survival of locally advanced LSCC patients between VATS and thoracotomy after neoadjuvant chemotherapy. The primary goal of this study was to explore the feasibility and safety of VATS following neoadjuvant chemotherapy for locally advanced LSCC in terms of intraoperative and postoperative outcomes.

## Methods

### Patients selection

The study protocol was approved by the Institutional Review Board of the First Affiliated Hospital of Zhejiang University, School of Medicine. The data in this study was collected retrospectively from hospital electronic medical records system, including demographic characteristics, preoperative investigations, intraoperative data and postoperative course between October 2013 and October 2017.

All patients included in the analysis were restaged by AJCC 8 staging system [14] and fitted the following criteria: (1) the disease was pathologically diagnosed as LSCC; (2) the patient did not have distant metastasis before neoadjuvant chemotherapy; (3) the surgery was preceded by neoadjuvant chemotherapy.

We excluded patients with a history of previous cancers, other concurrent malignant disease and patients who underwent pulmonary resection previously. Locally advanced squamous lung cancer was mainly defined as stageIII, while the patients with stage T3 was also regarded as locally advanced disease. Clinical lymph node (LN) status was assessed by CT scan, PET scan and/or endobronchial ultrasound. Tumor size was defined as the maximum diameter of the pathological specimens.

Patients were retrospectively classified into the thoracotomy group and VATS group on the basis of the surgical approach. Patients undergoing conversion in the VATS group were eliminated from the study group.

### Treatment protocol and response assessment

Neoadjuvant chemotherapy consisted of platinum-based two-drug regimen with 2 cycles, while the cycle was adjusted with tumor response and adverse effects after systematic evaluation. Generally, the resection was performed within 6 weeks after neoadjuvant chemotherapy and adjuvant therapy was carried out depending on the recovery condition of patients. Tumor response was classified according to Response Evaluation Criteria in Solid Tumors version 1.1 (RECIST 1.1) criteria [15]. The patients who had received at least 1 cycle of chemotherapy were candidates for response assessment.

### Surgical procedures

All patients underwent general anesthesia with single-lung ventilation and were placed in lateral decubitus position. Conventional posterolateral serratus divided thoracotomies were performed in the open procedures, and 3-ports approach was adopted in the thoracoscopic procedures. Generally, bronchi, pulmonary vasculature and parenchyma were resected by the corresponding endoscopic cut stapler. Prior closing, the cavity was rinsed by normal saline to detect potential air leak and one chest tube was placed in the appropriate position at the end of the procedure. The tube was removed when it was clearly confirmed no air leak and the volume of drainage was less than 200 mL/day. In contrast, when pneumonectomy was performed the tube was normally clipped after surgery and removed when there was no abnormal appearance in roentgenograms.

### Follow-up

Follow-up data were collected by telephone calls and reviewing the records of reexamination in the outpatient clinic. The last follow-up time was February 2018. The outcomes of interest of the current study included disease-free survival (DFS) and overall survival (OS). DFS was calculated from the day of surgery to the date of cancer recurrence or death from any cause. Patients who did not have a recurrence or who did not die during the study period were censored at the date they were last confirmed to be alive with no evidence of disease. OS was calculated from the day of surgery to the time of death. Patients who did not die during the study period were censored at the date they were last confirmed to be alive.

### Statistical analysis

The measurement data and numeration data were statistically analyzed with t test and χ^2^ test respectively. DFS and OS were estimated using the Kaplan-Meier method. All the above analysis was conducted by SPSS software (version 19.0, IBM SPSS Inc. United States). The statistical power analysis was further conducted by R (version 3.2.5; R Development Core Team) when the differences between the two groups were statistical significant. Statistical significance was set at *P* value < 0.05 (All *P* values presented were 2-sided).

## Result

### Patients’ characteristics

From October 2013 to October 2017, a total of 83 patients fitted the criteria for inclusion in the study: 67 treated with thoracotomy and 16 treated with VATS. Two patients converted to thoracotomy because of severe adhesions were eliminated from the VATS group, so there were 67 patients in the thoracotomy group and 14 patients in the VATS group finally. The major demographic and clinical characteristics were listed in Table 1. There was no significant gender difference between thoracotomy group and VATS group, in which the male gender occupied 94.0 and 78.6%, respectively. The age was also similar between two groups. It was comparable in the two groups for the number of patients with other possible prognostic factors which might be predictive of survival, including body mass index (BMI) [16], weight loss (more than 5%) [17] and other nutritional status [18].

**Table 1:** The demographic and clinical characteristics in the thoracotomy and VATS group

Variables	Thoracotomy (*N* = 67)	VATS (*N* = 14)	*P* value
Male gender	63 (94.0%)	11 (78.6%)	0.177
Age (year)	60 (29–77)	61 (55–73)	0.182
Smoking	59 (88.1%)	11 (78.6%)	0.608
Drinking	29 (43.3%)	5 (35.7%)	0.602
BMI	23 (17–30)	23 (18–29)	1
Weight loss	15 (22.4%)	2 (14.3%)	0.752
Hypertension	12 (17.9%)	6 (42.9%)	0.091
Diabetes	5 (7.5%)	1 (7.1%)	1
COPD	1 (1.5%)	0 (0)	1
Lymphocyte (10^9/L)	1.5 (0.8–4.0)	1.6 (0.7–3.2)	0.954
Total protein (g/L)	68.8 (54.4–82.4)	67.3 (39.2–80.6)	0.077
Albumin (g/L)	41.6 (26.6–54.8)	39.2 (20.9–50.9)	0.128
Serum Creatinine (mmol/L)	69.0 (52.0–105.0)	68.5 (50.0–119.0)	0.507
Serum trioxypurine (mmol/L)	296.5 (192.0–455.0)	319.5 (157.0–462.0)	0.676
Triglyceride (mmol/L)	1.1 (0.4–4.1)	1.2 (0.7–3.0)	0.581
Cholesterol (mmol/L)	4.2 (2.7–6.1)	4.3 (2.7–8.0)	0.399

Values are N (percentage) or median (range)

*BMI* body mass index, *COPD* chronic obstructive pulmonary disease, *VATS* video assisted thoracic surgery

### Disease characteristics

The disease characteristics of two groups were listed in Table 2 in detail. There was no significant difference between thoracotomy group and VATS group in the clinical stage before neoadjuvant chemotherapy with 54 (80.6%) and 13 (92.9%) patients in stage IIIA or IIIB, respectively. It was worth mentioning that 14 patients (13 in thoracotomy group and 1 in VATS group) with IIIA disease in seventh AJCC staging system were restaged as IIB disease in eighth AJCC staging system. More than half of the patients received the regimens consisted of gemcitabine and platinum with 2 cycles in both groups. T stage was similar in two groups either before neoadjuvant chemotherapy of after, as well as the number of T downstaging. Clinical complete response (cCR) was seen in 1 patient in both groups, retrospectively, while 43 (64.2%) patients in thoracotomy group and 11 (78.6%) patients in VATS group were evaluated as partial response (PR). Only 4 (6.0%) patients in thoracotomy group were classified in progressive disease (PD), with one patient having oligometastasis in 11th thoracic vertebra. However, the primary tumor of the patient with oligometastasis was detected with only few cancer cells under the microscope. The number of patients with downstaging after neoadjuvant chemotherapy which was a certain prognostic factor was comparable in two groups. There was also no statistical difference in pathologic stage and tumor size between two groups.

**Table 2:** The disease characteristics in the thoracotomy and VATS group

Variables	Thoracotomy (*N* = 67)	VATS (*N* = 14)	*P* value
T stage before neoadjuvant chemotherapy			0.135
1b	0 (0)	1 (7.1%)	
1c	1 (1.5%)	2 (14.3%)	
2a	25 (37.3%)	3 (21.4%)	
2b	7 (10.4%)	2 (14.3%)	
3	19 (28.4%)	3 (21.4%)	
4	15 (22.4%)	3 (21.4%)	
Clinical stage before neoadjuvant chemotherapy			0.508
IIB	13 (19.4%)	1 (7.1%)	
IIIA	32 (47.8%)	7 (50.0%)	
IIIB	22 (32.8%)	6 (42.9%)	
Neoadjuvant chemotherapy regimens			0.664
Docetaxel + platinum	16 (23.9%)	2 (14.3%)	
Paclitaxel + platinum	16 (23.9%)	3 (21.4%)	
Gemcitabine + platinum	35 (52.2%)	9 (64.3%)	
Cycles of neoadjuvant chemotherapy	2 (1–5)	2 (2–4)	0.930
T stage after neoadjuvant chemotherapy			0.058
0	1 (1.5%)	1 (7.1%)	
1b	1 (1.5%)	2 (14.3%)	
1c	0 (0)	1 (7.1%)	
2a	42 (62.7%)	8 (57.1%)	
2b	6 (9.0%)	0 (0)	
3	14 (20.9%)	1 (7.1%)	
4	3 (4.5%)	1 (7.1%)	
Clinical stage after neoadjuvant chemotherapy			0.308
0	1 (1.5%)	1 (7.1%)	
IB	15 (22.4%)	4 (28.6%)	
IIA	1 (1.5%)	0 (0)	
IIB	12 (17.9%)	0 (0)	
IIIA	25 (37.3%)	7 (50.0%)	
IIIB	12 (17.9%)	2 (14.3%)	
VI	1 (1.5%)	0 (0)	
Response assessment			0.261
cCR	1 (1.5%)	1 (7.1%)	
PR	43 (64.2%)	11 (78.6%)	
SD	19 (28.4%)	2 (14.3%)	
PD	4 (6.0%)	0 (0)	
T downstaging	28 (41.8%)	9 (64.3%)	0.124
TNM downstaging	29 (43.3%)	8 (57.1%)	0.344
Pathologic stage			0.221
IA	1 (1.5%)	3 (21.4%)	
IB	22 (32.8%)	3 (21.4%)	
IIA	2 (3.0%)	0 (0)	
IIB	18 (26.9%)	3 (21.4%)	
IIIA	18 (26.9%)	4 (28.6%)	
IIIB	5 (7.5%)	1 (7.1%)	
VI	1 (1.5%)	0 (0)	
Tumor size (cm)^a^	3.1 (0.8–8.0)	2.5 (1.0–7.0)	0.335

Values are N (percentage) or median (range)

*cCR* clinical complete response, *PR* partial response, *SD* stable disease, *PD* progressive disease

^a^Only few cancer cells in 4 patients were observed microscopically (3 patients in thoracotomy group and 1 in VATS group), so the tumor size of the 4 cases was unable to measure

### Perioperative data

The detailed information of surgical outcome was presented in Table 3. Surgical procedure was comparable in both groups, although there was no double sleeve lobectomy and pneumonectomy in VATS group. In contrast, the number of resected lymph nodes in thoracotomy group was more than that in VATS group (*P* = 0.011, power = 80.4%), but there was no significant difference in the number of resected lymph node stations and the case of nodal upstaging. The rate of negative surgical margin in VATS group reached up to 92.9% which was seemed to be higher than that in thoracotomy group but the difference was not of statistically significance. The operation time and blood loss were also similar in two groups, but the volume of chest drainage in VATS group was less than that in thoracotomy group (*P* = 0.019, power = 80.1%), although the duration of chest drainage was comparable. In addition, the VATS group had a clear advantage over the thoracotomy group in terms of postoperative pain (*P* = 0.005, power = 62.2%) which was recorded by numerical rating scale (NRS) [19]. Perioperative complications ranked by Clavien-Dindo classification [20] were comparable in thoracotomy and VATS groups with 9 (13.4%), 9 (13.4%), 2 (3.0%) and 1 (7.1%), 2 (14.3%), 0 (0) in GradeI, GradeII, GradeIII, respectively. The length of postoperative hospital stay was also comparable in two groups. The delay and protocol of adjuvant therapy after surgery in thoracotomy group were similar to those in VATS group as well.

**Table 3:** Perioperative data in the thoracotomy and VATS group

Variables	Thoracotomy (*N* = 69)	VATS (*N* = 14)	*P* value	Cohen’s d value	Statistical power
Surgical procedure			0.078		
Sublobar resection	1 (1.5%)	2 (14.3%)			
Lobectomy	39 (58.2%)	8 (57.1%)			
Bilobectomy	6 (9.0%)	3 (21.4%)			
Sleeve lobectomy	9 (13.4%)	1 (7.1%)			
Double sleeve lobectomy	2 (3.0%)	0 (0)			
Pneumonectomy	10 (14.9%)	0 (0)			
Surgical margin			0.760		
R0	59 (88.1%)	13 (92.9%)			
R1	7 (10.4%)	1 (7.1%)			
R2	1 (1.5%)	0 (0)			
Number of resected LNs	20 (2–57)	16 (1–28)	0.011	0.838	80.4%
Number of resected LN stations	7 (2–12)	7 (1–10)	0.856		
Nodal upstaging	13 (19.4%)	4 (28.6%)	0.685		
Operation time (minutes)	146 (87–410)	145 (73–364)	0.411		
Blood loss (ml)	100 (20–400)	83 (10–500)	0.819		
Numerical pain rating scale	2 (1–7)	2 (1–3)	0.005	0.676	62.2%
Chest drainage (ml)	1035 (150–5850)	550 (30–2100)	0.019	0.835	80.1%
Duration of chest drainage (days)	5 (2–20)	4 (2–15)	0.285		
Complications	20 (29.9%)	3 (21.4%)	0.729		
GradeI	9 (13.4%)	1 (7.1%)			
GradeII	9 (13.4%)	2 (14.3%)			
GradeIII	2 (3.0%)	0 (0)			
Postoperative hospital stay (days)	7 (4–21)	6 (4–16)	0.066		
Mortality within 30 days	0 (0)	0 (0)	/		
Delay of adjuvant therapy (days)	40 (21–127)	36 (23–57)	0.353		
Protocol of Adjuvant therapy			0.275		
Chemotherapy	36 (53.7%)	10 (71.4%)			
Radiotherapy	6 (9.0%)	0 (0)			
Chemoradiotherapy	16 (23.9%)	4 (28.6%)			
Unknown	9 (13.4%)	0 (0)			

Values are N (percentage) or median (range)

*LN* lymph node

### Survival outcome

**Fig. 1:**
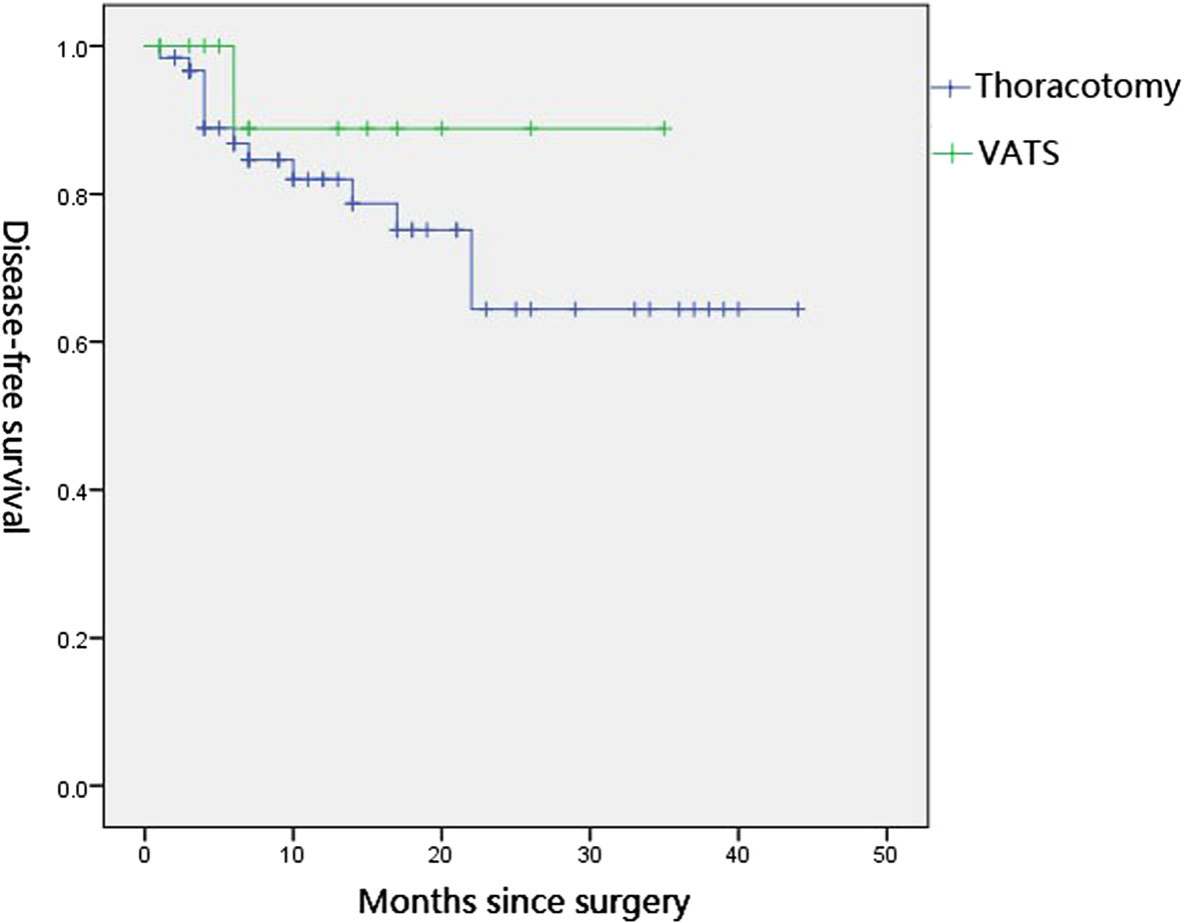
Kaplan-Meier curves for the disease-free survival of thoracotomy and VATS (*P* = 0.335)

**Fig. 2:**
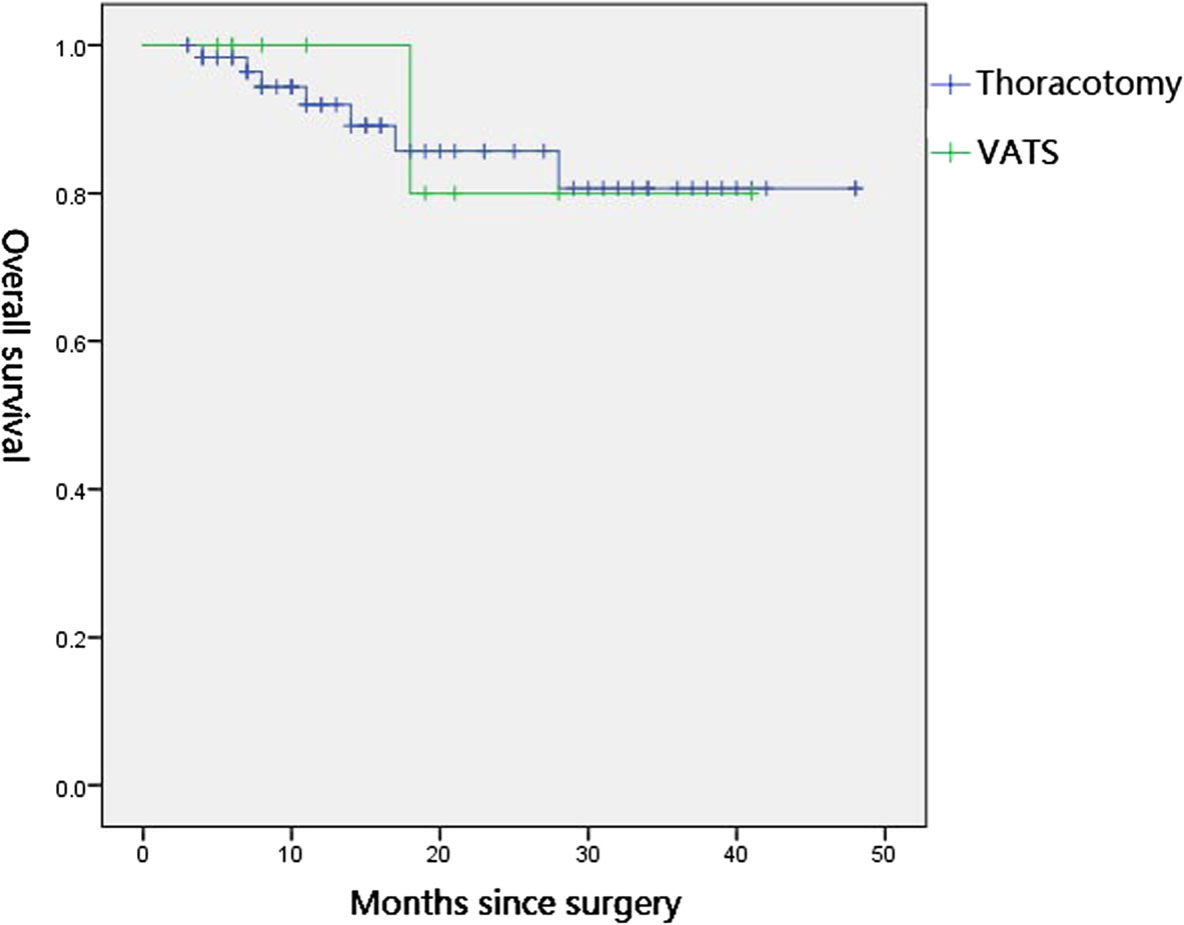
Kaplan-Meier curves for the overall survival of thoracotomy and VATS (*P* = 0.925)

Follow-up information was successfully collected from 73 of 81 patients with median follow-up time of 15 months (range: 3 to 48 months). Tumor recurrence and death occurred in 18 cases (9 deaths, 9 alive with disease). Four patients evaluated as PD after neoadjuvant chemotherapy were all alive during the follow-up period. The patient who had oligometastasis in 11th thoracic vertebra received concurrent chemoradiotherapy and had been living for more than 3 months after surgery with satisfactory life quality. Other patients had been living for 9, 13 and 16 months after surgery, respectively, with one patient alive with disease. The mean DFS and OS were 32.7 ± 2.7 months, 41.7 ± 2.2 months for the thoracotomy group and 31.8 ± 3.0 months, 36.4 ± 4.1 months for the VATS group, respectively. The differences between two groups were not statistically significant (Figs. 1 and 2).

## Discussion

In spite of the increased prevalence of early stage NSCLC with satisfactory survival outcome, the treatment of LANSCLC remains challenging. Early stage NSCLC patients are commonly treated with radical resection, but unfortunately most patients with LANSCLC do not benefit clearly by surgery alone or even by chemoradiotherapy [21]. In the result, neoadjuvant therapy has been proposed in order to better achieve local and distant disease control in LANSCLC. Although neoadjuvant chemoradiotherapy followed by surgery had been proven to be a feasible and safe treatment strategy [22, 23], several subsequent studies reported that preoperative radiotherapy significantly increased the occurrence of bronchopleural fistula after surgery [24] and did not add any survival benefit to neoadjuvant chemotherapy followed by surgery [7, 25]. Of course, neoadjuvant chemotherapy had some controversial problems, one of which was the challenges in surgery, including perioperative complications, interval time and the surgical approach.

In spite of lacking multicenter prospective researches, a large number of retrospective studies proved that neoadjuvant chemotherapy did not add any extra risk to the occurrence of perioperative complications and mortality [7, 26, 27] even though mediastinal structures become differently affected after neoadjuvant chemotherapy. In addition, the optimal interval time from the end of neoadjuvant chemotherapy to surgery was proven to be not more than 6 weeks [28]. However, there was little evidence suggesting the preferable surgical approach after neoadjuvant chemotherapy in LANSCLC, especially in local advanced LSCC, thoracotomy or VATS. Historically, VATS was initially recommended only for early stage disease, but with the technological improvement and growing experience, a few experts started to use the VATS platform to carry out pulmonary resection in LANSCLC. Jun Huang et al. [13] publishing a single institution retrospective series of 43 cases reported that VATS following neoadjuvant therapy was safe and feasible for the treatment of LANSCLC with low incidence of postoperative complications and mortality. Unfortunately, this study did not compare the surgical and survival outcomes of VATS with those of thoracotomy. Another study reported by Bernard J. Park et al. [12] compared minimally invasive lobectomy (VATS and robotic) with open lobectomy and concluded that minimally invasive surgery possessed good feasibility, good safety and an acceptable survival time in appropriately selected patients with LANSCLC after neoadjuvant chemotherapy. However, this study mainly focused on lung adenocarcinoma and lobectomy. Meanwhile, the age between two groups had statistical difference, which might cause potential bias in the conclusion.

In this study, we explored whether VATS was suitable to be applied in locally advanced LSCC staged by AJCC 8 staging system or not. We observed similar operative time and blood loss between VATS and thoracotomy, but VATS had advantages in postoperative pain and chest drainage. Interestingly, duration of chest drainage was similar in two groups, but the length of postoperative hospital stay was seemed to be shorter in VATS group although the difference was not statistically significant. The rate and the severity of complications were also comparable. These results suggested that VATS played a positive role in enhanced recovery after surgery (ERAS) in locally advanced LSCC with equivalent survival to thoracotomy.

Systematic lymph node resection is an important part of surgical treatment for LANSCLC. It is a controversial problem about the quality of nodal assessment provided by VATS when compared to thoracotomy and its impact on long-term survival. Some publications indicated more lymph nodes were resected and a higher nodal upstaging rate was found in open surgery [29], while other studies suggested no correlation between the surgical approach and the number of lymph nodes resected [30]. In this study, we thoroughly analyzed the quality of lymph node resection between VATS and thoracotomy after neoadjuvant chemotherapy. Although the number of resected lymph node in VATS group was less than that in thoracotomy group, the number of resected stations and the rate of nodal upstaging were both similar in two groups. It suggested that a radical lymph node dissection could be achieved by VATS following neoadjuvant chemotherapy. It was worthwhile to note that the rate of negative margin of VATS was equivalent to that of thoracotomy.

However, there are some limits in this study. First, it is the retrospective study that has unavoidable selected bias, so further prospective evidence is warranted to verify the validity of our findings. Second, this study included some patients restaged IIB disease because of the difference between the seventh AJCC staging system and the eighth in the T stage, but most patients were diagnosed as stage IIIA or IIIB. Lastly, the sample is relatively small in this study, although the statistical power analysis was further conducted when the differences between the two groups were statistically significant.

## Conclusions

This study suggested that in selected patients with locally advanced LSCC VATS following neoadjuvant chemotherapy played a positive role in ERAS and associated similar oncological and survival outcome with thoracotomy.

## Abbrevations

LSCC: Lung squamous cell carcinoma
LA: Lung adenocarcinoma
NSCLC: Non-small cell lung cancer
LANSCLC: Locally advanced non-small cell lung cancer
VATS: Video assisted thoracic surgery
DFS: Disease free survival
OS: Overall survival
AJCC 8: The eighth American Joint Committee on Cancer
LN: Lymph node
BMI: Body mass index
cCR: Clinical complete response
PR: Partial response
SD: Stable disease
PD: Progressive disease
ERAS: Enhanced recovery after surgery
COPD: Chronic obstructive pulmonary disease
